# Factorial invariance of the generalized anxiety disorder scale (GAD-7) in Latin America and the Caribbean

**DOI:** 10.3389/fpsyt.2025.1529424

**Published:** 2025-01-30

**Authors:** Norman López, Juan-Carlos Coronado, Daniela Ripoll-Córdoba, Nicole Caldichoury, César Quispe-Ayala, Breiner Morales-Asencio, Raúl Quincho-Apumayta, Cesar Castellanos, Juan Martínez, Juan Cárdenas-Valverde, Luis Mario Castellanos-Alvarenga, David Salazar, Irina Flores-Poma, Jorge Herrera-Pino, Wendy Bada, Yuliana Flórez, Karen Alcos-Flores, Boris Zurita-Cueva, Elsa Muñoz Romero, Cristian Romo, Regulo Antezana, Claudio Avila Saldaña, Pascual A. Gargiulo

**Affiliations:** ^1^ Escuela de Kinesiología, Facultad de Salud, Universidad Santo Tomas, Temuco, Chile; ^2^ Departamento de Ciencias Sociales, Universidad de la Costa, Barranquilla, Colombia; ^3^ Departamento de Procesos Terapéuticos, Facultad de Ciencias de la Salud, Universidad Católica de Temuco, Temuco, Chile; ^4^ Departamento de Ciencias Sociales, Universidad de Los Lagos, Osorno, Chile; ^5^ Universidad Nacional de Huancavelica, Huancavelica, Peru; ^6^ Instituto Dominicano para el Estudio de la Salud Integral y la Psicología Aplicada (IDESIP), Santo Domingo, Dominican Republic; ^7^ Graduate School of Education, Ana G. Mendez University, Caguas, Puerto Rico; ^8^ Universidad César Vallejo, Lima, Peru; ^9^ Escuela de Psicología, Facultad de Ciencias Sociales y Comunicaciones, Universidad Santo Tomás, Temuco, Chile; ^10^ Facultad de Educación, Universidad Nacional Daniel Alcides Carrión, Cerro de Pasco, Peru; ^11^ College of Medicine, Florida International University, Miami, FL, United States; ^12^ Departamento Académico de Pedagogía y Humanidades, Facultad de Educación Intercultural y Humanidades, Universidad Nacional Intercultural de la Amazonia, Pucallpa, Peru; ^13^ Departamento de Neurocirugía, Omni Hospital, Guayaquil, Ecuador; ^14^ Departamento de Ciencias Exactas, Universidad de Los Lagos, Osorno, Chile; ^15^ Departamento de Ciencias de la Actividad Física, Universidad de Los Lagos, Puerto Montt, Chile; ^16^ Laboratorio de Neurociencias y Psicología Experimental, Área de Farmacología, Facultad de Ciencias Médicas, Universidad Nacional de Cuyo, Mendoza, Argentina

**Keywords:** generalized anxiety disorder, psychometric indicators, invariance, test, Latin American population

## Abstract

**Introduction:**

The prevalence of Generalized Anxiety Disorder (GAD) has increased rapidly, highlighting the importance of its detection using quick tools applicable to men and women from different countries.

**Objective:**

To analyze the psychometric properties of the Generalized Anxiety Disorder Test (GAD-7) by gender and country in Latin America and the Caribbean (LAC).

**Method:**

A cross-sectional e-health study with 12,124 participants from 15 LAC countries (54.32% women, 45.68% men) was conducted, including participants from Argentina (7.3%), Bolivia (6.7%), Colombia (10.3%), Chile (6.9%), Costa Rica (4.9%), El Salvador (5.7%), Ecuador (7.2%), Guatemala (4.7%), Panama (5.1%), Paraguay (5.7%), Peru (8.6%), Puerto Rico (5.8%), the Dominican Republic (6.6%), Uruguay (6.3%), and Venezuela (8.2%). All participants completed the GAD-7 scale digitally.

**Results:**

A unidimensional structure of the GAD-7 was confirmed, explaining 70% of the variance. The model fit indices were adequate (RMSEA = 0.062; CFI = 0.997; TLI = 0.995; SRMR = 0.017; p < 0.001), and the factor loadings for each item were satisfactory (> 0.70). Additionally, the factor structure showed measurement invariance between genders and countries, with adequate fit indices at all levels (configural, metric, scalar, and strict), suggesting that the measurements are equivalent in both contexts. Finally, the internal consistency of the GAD-7 was high, with a McDonald’s Omega coefficient of 0.91.

**Conclusions:**

The GAD-7 exhibits a factor structure that is equivalent across genders and countries, demonstrating its validity and reliability for the rapid detection of GAD symptoms in different countries within the region.

## 1 Introduction

The COVID-19 pandemic had negative consequences on the mental health of the general population; especially generalized anxiety disorder, whose post-pandemic prevalence increased by 25% (1). Clinically, GAD is expressed through excessive worry (anxious anticipation) about various events or activities. The intensity, duration, or frequency of GAD is often disproportionate to the actual likelihood or impact of an event (2).

Scientific evidence indicates that this disorder is closely linked to intense and difficult-to-manage stress situations. In many cases, worsening anxiety can lead to the onset of depressive symptoms (3); representing a clear indicator of psychological distress (4), with negative effects on both mental and physical health, and a direct impact on daily activities (5).

Additionally, anxiety often coexists with other disorders, such as depression, sleep disorders (6), and suicidal ideation (7, 8), acting as a trigger for multiple diseases (9, 10). This comorbidity not only intensifies anxiety symptoms but also prolongs their duration and reduces the effectiveness of interventions (11). Therefore, early detection is essential to mitigate its impact on mental health and develop prevention and treatment plans that minimize the occurrence of associated pathologies (12), alleviating the burden on the individual and facilitating recovery (3).

An economical, valid, and reliable way to detect GAD is through rapid tests. The Generalized Anxiety Disorder Test (GAD-7) (13), is a self-administered clinical scale used to assess generalized anxiety disorder over the past two weeks, according to DSM-V criteria (2). It is an instrument that is easy to apply, score, and interpret, with widespread use in hospital systems and strong support in the medical literature (14, 15).

The GAD-7 has demonstrated good clinical utility and excellent psychometric properties for quickly assessing GAD symptoms in international studies, showing adequate internal consistency (Cronbach’s Alpha >.70) (16, 17). During the COVID-19 pandemic, its use expanded to numerous studies worldwide (18–20). In Latin America and the Caribbean (LAC), it has also been applied, showing robust validity and reliability indicators (15, 21).

However, to date, no studies have provided evidence of the GAD-7’s invariance based on demographic characteristics in this region. This information is essential as it would strengthen the instrument’s validity for use in diverse populations within LAC. Therefore, the objective of this study was to evaluate the factorial invariance of the Generalized Anxiety Disorder Test considering gender and country of residence in a large sample of adults from the region.

## 2 Materials and methods

### 2.1 Participants

An e-Health study (22) was conducted using a snowball sampling methodology. Through social networks, personal, and institutional emails, the general population aged 18 and older from 15 countries in the region was invited to complete an online questionnaire. To ensure proper coordination of the project, an international research consortium established interinstitutional agreements with hospitals, civil and governmental organizations, universities, and professional associations.

A logistical framework was developed to form working teams in each country, which were specifically trained for the study. Institutional email databases were compiled, and the online questionnaire was distributed via email, social networks, and WhatsApp, encouraging its dissemination within the communities. Data collection began on May 12, 2022, and concluded on November 27, 2023.

Initially, 14,842 participants were evaluated. However, 2,718 were excluded for not completing the questionnaire, not signing the informed consent, not reporting their gender, or selecting countries where an adequate number of forms could not be collected. As a result, 12,124 forms were processed (54.32% women and 45.68% men) from Argentina (7.3%), Bolivia (6.7%), Colombia (10.3%), Chile (6.9%), Costa Rica (4.9%), El Salvador (5.7%), Ecuador (7.2%), Guatemala (4.7%), Panama (5.1%), Paraguay (5.7%), Peru (8.6%), Puerto Rico (5.8%), Dominican Republic (6.6%), Uruguay (6.3%), and Venezuela (8.2%). The average age of the participants was 31.14 years (SD: 8.78). The final sample included health professionals (12.5%), engineers and exact sciences (9.3%), social sciences (8.2%), legal, accounting, and administrative sciences (10.3%), education sciences (11.4%), university students (14.2%), and community members (34.1%).

The complementary data table provides detailed demographic information about the participants, distributed by countries, age ranges, and the proportion of male and female participants (**Table 1**). It is worth noting that the representation of other genders was below 0.2%. Due to the lack of statistical power and representativeness of these groups, the analysis focused exclusively on the information provided by male and female participants.

**Table 1:** Complementary data table: demographic information of the participants.

Countries	No. subjects	% evaluations	Age ranges		% by gender	
			Lower Limit	Upper Superior	Male	Female
Argentina	885	7,3	21,78	39,31	49,96	50,04
Bolivia	812,3	6,7	25,5	43,06	43,74	56,26
Colombia	1249	10,3	24,76	42,32	48,68	51,32
Chile	836,5	6,9	21,76	39,32	47,73	52,27
Costa Rica	594	4,9	20,58	38,14	43,7	56,3
El Salvador	691	5,7	30,25	47,81	44,68	55,32
Ecuador	873	7,2	23,9	41,46	43,75	56,25
Guatemala	570	4,7	25,52	43,08	53,16	53,16
Panamá	618,3	5,1	22,27	39,83	45,77	54,23
Paraguay	691	5,7	20,6	38,16	43,99	56,01
Perú	1042,8	8,6	18,84	36,4	43,67	55,33
Puerto Rico	703,1	5,8	17,72	35,28	47,34	52,66
República Dominicana	800,1	6,6	19,49	37,05	44,69	55,31
Uruguay	763,8	6,3	23,66	41,22	46,92	53,08
Venezuela	994,1	8,2	18,83	36,39	42,74	57,26
Total	12124	100			46,03	54,32

### 2.2 Instruments

An automated Google form was used, which provided information about the study’s objective, informed consent, along with demographic questions. Additionally, the GAD-7 was applied, which, through seven questions, allows for a quick assessment of the presence and severity of generalized anxiety disorder over the past two weeks (2, 13). The score ranges from 0 to 3 for each item, with response options being “Not at all,” “Several days,” “More than half the days,” and “Nearly every day.” The total GAD-7 score can range from 0 to 21, with a score of ≥10 indicating generalized anxiety disorder. It also allows for grading the severity of the disorder.

### 2.3 Data analysis

The data were digitized using Google Forms in a Google Spreadsheet. The database was downloaded as an xlsx file and imported into R software version 4.02 in its RStudio programming environment version 1.3.595 (23). The openxlsx (24) package was used for data import, and the tidyverse (25) and psych (26) packages were used for data preparation and analysis. The lavaan (27), semPlot (28), and semTools (29) packages were used for confirmatory factor analysis (CFA) and measurement invariance. The MBESS (30) packages were used for confirmatory factor analysis (CFA) and measurement invariance. The MBESS (31) package was used to calculate the winsorized correlation coefficient (tr. = 0.10). For the CFA, the Robust Weighted Least Squares Mean and Variance adjusted (WLSMV) estimator was used, and unidimensional structure was evaluated. For the evaluation of fit indices, the following criteria were considered: values ≥.90 and ≥.95 for CFI and TLI, as adequate and good fit, respectively; values ≤.08 and ≤.05 for RMSEA, as adequate and good fit, respectively; and for SRMR, values ≤.08 and ≤.06 were considered good and ideal fit, respectively. The decision to apply measurement invariance analysis instead of Differential Item Functioning (DIF) was based on the study’s objective to compare latent structures across different groups. While DIF analysis focuses on detecting item-level biases, measurement invariance evaluates whether the overall factorial structure of a scale is comparable across groups, ensuring that observed differences reflect true variations in the latent construct rather than measurement biases. This approach was deemed more suitable for evaluating the invariance of the psychometric model across gender and country groups, following the procedures proposed by Wu & Estabrook (31), and using established cutoff criteria (ΔCFI <.010, ΔTLI <.010, ΔSRMR <.030, and ΔRMSEA <.015) to determine invariance levels (32–34).

### 2.4 Ethical considerations

The study adhered to the ethical standards of the relevant national and institutional committees and the Declaration of Helsinki of 1975, as revised in 2008. Informed consent was obtained online from all participants, who, upon completing the assessment, received a report of their results and a document containing psychological and clinical guidance. The protocol was approved by the Ethics Committee of Universidad de La Costa (Record No. 173 of May 27, 2024, research project code INV. 140-03-001-18).

## 3 Results

In **Table 2**, the descriptive statistics for the seven items of GAD are shown. It can be observed that item 5 has the lowest mean value, while item 3 has the highest mean value. Regarding Skewness and Kurtosis, these are close to zero, indicating that the data distribution is unlikely to affect subsequent analyses.

**Table 2:** Descriptive statistics for the GAD-7 Items.

N item	*M*	*SD*	Skewness	Kurtosis
1. Feeling nervous, anxious, or on edge.	1.34	0.91	0.25	-0.71
2. Not being able to stop or control worrying/restlessness.	1.11	0.88	0.46	-0.48
3. Feeling very restless about different things.	1.41	0.91	0.07	-0.82
4. Having trouble relaxing.	1.52	0.94	0.07	-0.90
5. Being so restless that it is difficult to sit still.	1.08	0.88	0.45	-0.54
6. Getting angry or irritated easily.	1.37	0.93	0.24	-0.78
7. Feeling afraid as if something terrible is going to happen.	1.30	1.00	0.28	-0.99

Next, confirmatory factor analysis was conducted to study the internal structure of the GAD-7. The models analyzed and their fit indices are presented in **Table 3**. For the instrument, the unidimensional structure without correlated errors showed a good fit in the CFI, TLI, and SRMR indices. However, the RMSEA index indicated an inadequate fit; therefore, the model was re-specified by adding the covariance between item 1 and item 2, which reduced the RMSEA value, indicating an adequate fit (<.08) to the model. Moreover, a theoretical explanation is that GAD-7 items 1 and 2 measure closely related constructs within the domain of generalized anxiety. Item 1 (“Feeling nervous, anxious, or on edge”) and item 2 (“Not being able to stop or control worrying/disquiet”) reflect a common core of anxiety-related emotional arousal and intrusive thoughts (32). Both items target the core of generalized anxiety disorder, which is characterized primarily by excessive worry and difficulties in controlling those thoughts (33). Therefore, it is expected that they share a considerable portion of common variance, which explains the need to model this covariance.

**Table 3:** Analyzed models and fit indices.

Instrument	Models	χ²	gl	CFI	TLI	RMSEA	SRMR
GAD	Model 0	61,310.24	21	.993	.989	.093	.023
	Model with covariance of errors between items 1 and 2	61,310.24	21	.997	.995	.062	.017

CFI, Comparative Fit Index; TLI, Tucker-Lewis Index; RMSEA, Root Mean Square Error of Approximation; SRMR, Standardized Root Mean Square Residual.

**Figure 1:**
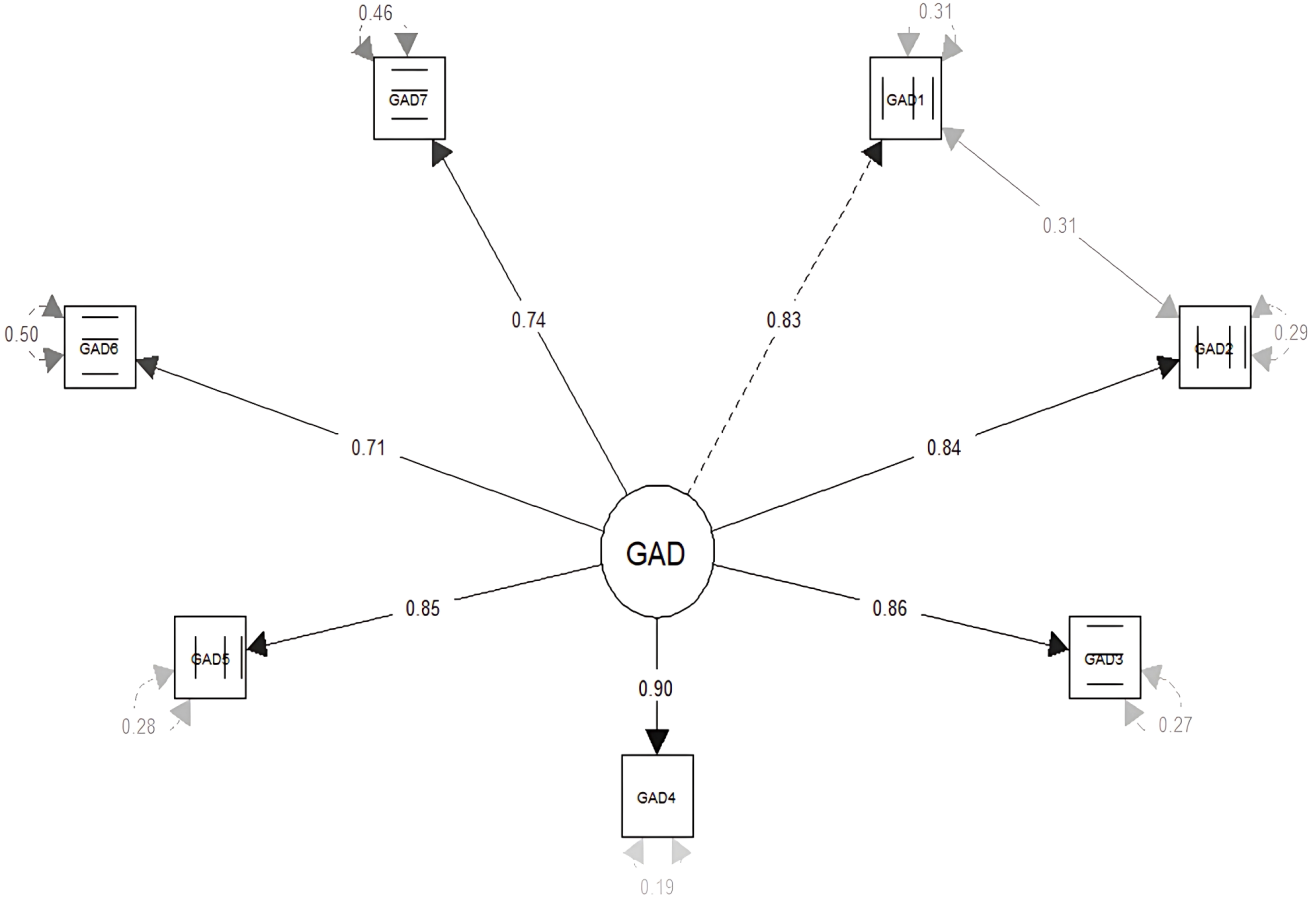
Factor loadings and correlated errors of the GAD-7.

**Figure 1** presents the factor loadings of the model that showed the best fit for the GAD. In this case, the factor loadings were greater than.71, and the correlated error between item 1 and item 2 had a value of.31.

Subsequently, the invariance of the GAD-7 measures was evaluated according to gender and country. As shown in **Table 4**, the invariance analysis reveals that the factor structure of the GAD-7 by gender showed fit indices ranging from adequate to good at the configural, threshold, metric, scalar, and strict levels. Additionally, the differences between the fit indices were smaller than the value established in the data analysis section, indicating that the factor structure of the instrument presents measurement invariance and is equivalent for both groups (men and women). The same results were observed when using the country as the comparison variable. It was found that the structure (configural), thresholds, factor loadings (metric), intercepts (scalar), and residuals (strict) were equivalent between the group.

**Table 4:** Measurement invariance by gender and country.

	Model	Χ^2^	Gl	CFI	TLI	RMSEA	SRMR	ΔCFI	ΔTLI	ΔRMSEA	ΔSRMR
**GAD**	Gender										
	Configural	249.443	26	.996	.994	.068	.019	–	–	–	–
	Threshold	275.190	33	.996	.995	.063	.019	.000	.001	-.005	.000
	Metric	309.855	39	.996	.995	.061	.020	.000	.000	-.002	.000
	Scalar	303.143	45	.996	.996	.056	.020	.000	.001	-.006	.000
	Strict	358.150	52	.995	.996	.056	.025	-.001	.000	.001	.005
	Country										
	Configural	362.541	78	.995	.992	.078	.023	–	–	–	–
	Threshold	424.877	113	.995	.994	.068	.023	.000	.002	-.010	.000
	Metric	440.097	143	.995	.996	.059	.023	.000	.001	-.009	.001
	Scalar	485.082	173	.995	.996	.055	.023	.000	.001	-.004	.000
	Strict	699.466	208	.992	.995	.063	.037	-.003	-.001	.008	.013

Finally, McDonald’s Omega coefficient was applied to evaluate the internal consistency of the instrument. The value obtained for the GAD-7 test was ω = .91.

## 4 Discussion

We analyzed the factor structure, and the reliability level of the GAD-7 test to detect GAD symptoms, considering the concepts of gender and country invariance, in a large sample of the surveyed population from LAC.

Factor analysis revealed that the GAD-7 has a unidimensional structure, explaining 70% of the variance and demonstrating adequate fit indices (RMSEA = 0.062, CFI = 0.997, TLI = 0.995, SRMR = 0.017). This confirms that the items of the instrument reflect a single factor of generalized anxiety. This finding aligns with international literature, which has consistently shown in various national contexts that the GAD-7 reliably measures a single factor associated with disorder symptoms (13, 34). Furthermore, multiple global studies have validated its reliability, establishing it as a key tool for both clinical practice and research (17, 20, 35, 36).

These results indicate that the GAD-7 can identify symptoms of generalized anxiety without the need to divide the items into subscales. The consistency of its factorial structure across different countries supports the instrument’s validity and reinforces its applicability at an international level, offering a standardized approach to detecting the disorder. This is particularly relevant in Latin America and the Caribbean, where reliable, accurate, and affordable tools are needed for the consistent detection of generalized anxiety.

Secondly, we demonstrated the factorial invariance of the GAD-7 considering participants’ gender and country of residence. The results indicate that the GAD-7 maintains equivalence at all levels (configural, metric, scalar, and strict), both between men and women and across the different countries evaluated. These findings are particularly significant, given that studies analyzing the stability of the instrument by gender and country remain limited (36); although the invariance of the instrument has been confirmed in other contexts (37–39).

In this context, the available evidence has demonstrated the usefulness of the GAD-7 in various settings across Latin America. The instrument has been used to identify symptoms of generalized anxiety in populations receiving healthcare services (14, 40, 41), university students (42–44), and the general population (36, 45, 46).

Finally, our study confirmed the high reliability of the GAD-7, as evidenced by an Omega coefficient of 0.91, which ensures the instrument’s ability to consistently detect GAD symptoms. Specifically, a value greater than 0.90 reflects a high degree of homogeneity among the test items, ensuring that each item contributes equally to measuring the construct’s structure. These findings align with previous studies conducted in various countries, which have also reported high internal consistency indices for the GAD-7 (16, 47–50).

These findings are particularly relevant in the Latin American context, where access to mental health services is limited. In this scenario, the GAD-7 stands out for its reliability and stability in measurement, exceeding the recommended threshold for evaluations in clinical practice. This makes it a fast and accurate tool that facilitates early diagnosis and timely interventions for the detection of GAD.

## 5 Limitations

Although this study presents adequate results, it has some limitations. First, construct validity was not assessed; however, the analyses showed that the GAD-7 has adequate internal consistency and a factorial structure invariant across gender and country. Second, the cross-sectional design limits the evaluation of changes in generalized anxiety levels over time. Future longitudinal studies would be valuable, particularly in a post-pandemic context. Third, no information was collected on premorbid state or prior mental health conditions, making it difficult to conduct more detailed causal analyses, such as case-control studies. Fourth, although the sample included several countries from Latin America and the Caribbean (LAC), it lacked representation of minority subgroups, such as Indigenous communities, rural areas, and economically vulnerable populations. This limits the understanding of the cultural applicability of the GAD-7 and its cultural equivalence, which could be improved by expanding the sample (44, 45). Fifth, factorial invariance by age was not analyzed, a relevant aspect since anxiety symptoms may vary across age groups. Finally, the use of self-reported data poses risks of response bias and limits the generalization of results. The absence of clinical variables and diagnostic interviews restricts clinical validation. Advanced methodologies, such as Differential Item Functioning (DIF) analysis, would be necessary to address potential cultural biases and improve accuracy in diverse populations.

Currently, the scale validation is based on self-reported data, which is a widely used approach in psychological and social sciences research. However, future studies should incorporate clinical validation using criterion-related measures (51), such as structured interviews or comparisons with established diagnostic tools. The inclusion of external validation criteria would ensure that the scores obtained through the scale are aligned with clinically recognized constructs (52), thereby increasing the scale’s applicability in both research and clinical settings.

Future research should aim to validate the scale using clinical criteria, such as structured interviews and comparisons with diagnostic tools, to strengthen the interpretative validity of the results. The inclusion of clinical background information, such as medication use and comorbidity, would provide a more comprehensive understanding of the population. Additionally, employing Item Response Theory (IRT) models, such as the Rasch model (53, 54), and conducting cross-group comparisons by gender and country would enhance the generalizability and cultural sensitivity of the scale. Finally, addressing self-selection bias through random or stratified sampling techniques would reduce ambiguity concerning the population represented in the study and improve the inferential validity of the findings.

Considering these limitations, we can conclude that the GAD-7 maintains a stable factorial structure across both men and women, as well as among different countries in LAC. This provides a solid empirical foundation for the use of the GAD-7 in clinical and epidemiological research within the region and across diverse contexts. Additionally, it facilitates the early detection of anxiety and supports the implementation of mental health policies that address the specific needs of the region.

## Data Availability

The raw data supporting the conclusions of this article will be made available by the authors, without undue reservation.
